# Translating an early childhood obesity prevention program for local community implementation: a case study of the Melbourne InFANT Program

**DOI:** 10.1186/s12889-016-3361-x

**Published:** 2016-08-08

**Authors:** R. Laws, K. D. Hesketh, K. Ball, C. Cooper, K. Vrljic, K. J. Campbell

**Affiliations:** 1 Institute for Physical Activity and Nutrition, School of Exercise and Nutrition Science, Deakin University, Geelong, VIC Australia; 2 Centre for Obesity Management and Prevention Research Excellence in Primary Health Care (COMPaRE-PHC), Sydney, Australia; 3 Centre of Research Excellence in Early Prevention of Obesity in Childhood, Sydney, Australia; 4 Prevention and Population Health, Department of Health and Human Services, Melbourne, Australia

**Keywords:** Obesity prevention, Children, Infants, Implementation, Research translation, Dissemination

## Abstract

**Background:**

While there is a growing interest in the field of research translation, there are few published examples of public health interventions that have been effectively scaled up and implemented in the community. This paper provides a case study of the community-wide implementation of the Melbourne Infant, Feeding, Activity and Nutrition Trial (InFANT), an obesity prevention program for parents with infants aged 3–18 months. The study explored key factors influencing the translation of the Program into routine practice and the respective role of policy makers, researchers and implementers.

**Methods:**

Case studies were conducted of five of the eight prevention areas in Victoria, Australia who implemented the Program. Cases were selected on the basis of having implemented the Program for 6 months or more. Data were collected from January to June 2015 and included 18 individual interviews, one focus group and observation of two meetings. A total of 28 individuals, including research staff (*n* = 4), policy makers (*n* = 2) and implementers (*n* = 22), contributed to the data collected. Thematic analysis was conducted using cross case comparisons and key themes were verified through member checking.

**Results:**

Key facilitators of implementation included availability of a pre-packaged evidence based program addressing a community need, along with support and training provided by research staff to local implementers. Partnerships between researchers and policy makers facilitated initial program adoption, while local partnerships supported community implementation. Community partnerships were facilitated by local coordinators through alignment of program goals with existing policies and services. Workforce capacity for program delivery and administration was a challenge, largely overcome by embedding the Program into existing roles. Adapting the Program to fit local circumstance was critical for feasible and sustainable delivery, however balancing this with program fidelity was a critical issue. The lack of ongoing funding to support translation activities was a barrier for researchers continued involvement in community implementation.

**Conclusion:**

Policy makers, researchers and practitioners have important and complementary roles to play in supporting the translation of effective research interventions into practice. New avenues need to be explored to strengthen partnerships between researchers and end users to support the integration of effective public health research interventions into practice.

**Electronic supplementary material:**

The online version of this article (doi:10.1186/s12889-016-3361-x) contains supplementary material, which is available to authorized users.

## Background

It is widely accepted that the transfer of new knowledge from public health research into policy and practice is far from optimal. Government agencies, including the National Institute of Health in the USA emphasise the need for widespread dissemination of evidence-based interventions to help bridge the gap between research and practice [1]. There is a growing body of literature investigating the translation of public health research into practice [2–4]. Five main stages of building evidence in public health have been proposed: Stages one and two, *Problem definition* and *Solution generation* relate to program development; stage three, *intervention testing,* represents process and impact evaluation to determine program efficacy or effectiveness; and stage four, *intervention replication,* refers to subsequent studies in which effective programs are adapted for other settings to determine if and how similar outcomes can be achieved in different places and populations [5]. Finally stage five, *intervention dissemination,* focuses on the scaling up of an effective program to population level to maximise public health impact [5].

Translational research in public health has been defined as studies that focus on stages four and five, that is replication and scaling up of effective interventions [5]. Scaling up is the process by which health promotion interventions shown to be effective in controlled conditions or on a small scale are expanded into real world practice [6]. There is growing interest in the concept of ‘scaling up’; however existing literature to date has been limited in focus, for example, investigations of conceptual frameworks [6–8] or case studies of scaled up programs in low income countries [8–10]. There are relatively few examples of published studies reporting on the scaling up of effective public health interventions into practice [11–14].

In the emerging field of obesity prevention in young children much of the research conducted to date is in the early stages of intervention development and efficacy testing (stages one to three), with a lack of effective population wide programs, particularly in young children 0–5 years [15–18]. Further, reporting of external validity information such as selection and representativeness of settings, intervention characteristics and delivery costs and program sustainability is poor in existing intervention studies [17, 19] and in systematic reviews on the topic [20]. Given the extent of child obesity as a public health problem, there is currently limited practice-relevant information for policy makers and practitioners to inform decisions about how effective programs can be disseminated ‘at scale’.

This paper provides a case study of the scaling up of the Melbourne InFANT Program (herein referred to as the InFANT Program), a group-based obesity prevention program that was effective in improving child and maternal diet, parental feeding behaviours and sedentary behavior in children [21]. The aim of the study was to explore the key factors influencing the scaling up and translation of this program into routine practice from the perspective of the main players involved, including researchers, policy makers and implementers. This will provide much needed insights into the ‘scaling up’ process and key lessons for public health researchers, policy makers and practitioners to inform the dissemination of other obesity prevention programs targeting young children into practice.

### Study context – The InFANT Program

The InFANT Program was a cluster randomised controlled trial (RCT) targeting first time mothers through existing universal care services which trialled the efficacy of a low dose program (six sessions delivered by dietitians quarterly over 15 months, commencing when Infants were 3 months of age) to improve parent and child diet and physical activity and to reduce sedentary behaviours [22]. This study was informed by qualitative research with the target group [23] and by two systematic reviews [15, 24]. At follow up when children were 18 months old, the Program did not change children’s growth, but did improve aspects of child’s diet and sedentary behaviours [21]; improved child diet quality [25]; improved water and vegetable intakes in sub-groups [26]; increased maternal knowledge and improved preferred feeding behaviours [27]; and improved mother’s dietary patterns [28].

### Dissemination context

The Program was delivered as part of The Victorian Department of Health and Humans Services (referred to throughout as ‘the Department’) prevention platform taking a complex whole of systems approach to reducing chronic disease risk. This approach involves delivering multiple strategies, policies and initiatives at both the state and local levels to target individuals in places where they spend their time, including childcare centres, schools, workplaces, food outlets, sporting clubs, businesses, local governments, health professionals and more to create healthier environments for all. This has shifted action from projects, small in scale, to prevention at scale delivered by multiple stakeholders, organisations, and sectors together with a community led placed based approach in 12 prevention areas across the state. Prevention areas were generally defined by local government area boundaries, however in some cases prevention area incorporated more than one local government area.

High quality health promotion programs have long played a significant role in health promotion, and with an opportunity to implement a complex system approach to prevention in Victoria, an opportunity emerged to re-think how programs can be delivered to contribute to prevention system change. The Department provided the opportunity for the 12 prevention areas to select quality health promotion programs using a selection criteria developed by the Department (Additional file 1) to support this decision making process. A list of recommended programs was created, which included the InFANT Program to fast track and support this process.

### Dissemination process

The InFANT Program researchers were funded by The Department to prepare program materials for dissemination. This included a facilitator manual, a parent handbook, a program website (www.infantprogram.org) and a guide for program implementation. A one day training program was developed and delivered by InFANT Program research staff to facilitators. Facilitators included dietitians, Maternal and Child Health (MCH) nurses and parent support workers. MCH nurses in Victoria provide a universal free service to parents of children 0-6 years and parent support workers typically work alongside MCH nurses to facilitate group programs such as first time parent groups. Prevention area staff were responsible for coordinating the implementation of the Program in each locality in partnership with key stakeholders and delivery agents.

## Methods

A case study approach was used to explore factors influencing the translation of the InFANT Program into routine practice. Case study methods are appropriate for answering ‘how’ and ‘why’ questions and when the phenomenon of interest (research translation) is embedded within a real-world policy and practice context [29]. The study consisted of a case series of five areas implementing the InFANT Program along with interviews with research staff and policy makers involved in the translation efforts across sites.

The study methods were informed by a constructionist epistemology. This assumes that knowledge is constructed and shaped by people’s perception, that phenomenon can only be understood in the context in which they are studied, and that truth is a matter of consensus amongst informed constructions, not a correspondence with an objective reality [30]. This was applied to the study by collecting detailed information on contextual factors influencing participants’ perceptions and recognising that the findings are co-created by participants and researchers and not an objective truth to be discovered.

### Recruitment

The sampling frame for the study sites were prevention areas that had been implementing the Program for at least 6 months at the commencement of data collection. The 6 month timeframe for implementation was selected to allow areas to have had some experience of key implementation issues. As of January 2015, eight out of the 12 prevention areas choose to deliver the Program, five were eligible to participate in this study having implemented the Program for at least 6 months.

The sampling strategy was purposeful with the aim to obtain insight from a range of stakeholders in each site as well as researchers and policy makers (Table 1). Coordinators (*n* = 5) from these five areas were emailed by the Department personnel to invite them to participate in an individual telephone interview, and all agreed. Following each interview, coordinators were asked to pass on the interview invitation to key local stakeholders and program facilitators who they considered might offer additional insights into program implementation in their area. Local key stakeholders included MCH Nurse Managers, those working in roles within Child and Family Services, staff involved in hosting or organising program venues, administrative or evaluation staff. A total of 11 staff (seven key stakeholders and four facilitators) were invited and agreed to participate using this snowballing method (Table 1). In one area, this took the form of a focus group (as this was already planned as part of local level evaluation), in the remaining areas individual telephone interviews were conducted. An online survey of program facilitators was also used as a concurrent recruitment strategy. A total of eight program facilitators from the participating areas completed the survey, and all agreed to be interviewed including three who had not already been identified through the snowballing strategy. Research staff and policy personnel who were actively involved in the translation effort were also invited to participate by email invitation from the study lead (RL), and all except one (a policy maker who had moved on to a different role) agreed. Research staff included the lead investigator involved in the design and testing of the Program in the RCT, those involved in training program facilitators and liasing with with policy personnel and local implementers.

**Table 1:** Participant position, area, recruitment and data collection methods

Interviewee code	Position	Area	Recruitment method	Data collection method
Research staff				
1	Research Staff	n/a	Direct invitation	Individual interview
2a	Research Staff	n/a	Direct invitation	Individual interview
2b	Research Staff	n/a	n/a	Meeting 1
2c	Research Staff	n/a	n/a	Meeting 2
3	Research Staff	n/a	Direct invitation	Individual interview
4	Research Staff	n/a	Direct invitation	Individual interview
Implementers				
5a	Coordinator	1	Direct invitation	Individual interview
5b	Coordinator	1	n/a	Meeting 1
5c	Coordinator	1	n/a	Meeting 2
5d	Coordinator	1	Direct invitation	Focus group
6	Program Facilitator	1	Snowballing	Focus group
7	Program Facilitator	1	Snowballing	Focus group
8	Program Facilitator	1	Snowballing	Focus group
9	Key Stakeholder	1	Snowballing	Focus group
10	Key Stakeholder	1	Snowballing	Focus group
11	Key Stakeholder	1	Snowballing	Focus group
12	Key Stakeholder	1	Snowballing	Focus group
13	Key Stakeholder	1	n/a	Meeting 2
14a	Coordinator	2	Direct invitation	Individual interview
14b	Coordinator	2	n/a	Meeting 1
15	Program Facilitator	2	Facilitator survey	Individual interview
16	Program Facilitator	2	Facilitator survey	Individual interview
17a	Coordinator	3	Direct invitation	Individual interview
17b	Coordinator	3	n/a	Meeting 2
18	Key Stakeholder	3	Snowballing	Individual interview
19	Key Stakeholder	3	Snowballing	Individual interview
20	Program Facilitator	3	Snowballing	Individual interview
21	Key Stakeholder	3	n/a	Meeting 1
22a	Coordinator	4	Direct invitation	Individual interview
22b	Coordinator	4	n/a	Meeting 1
23	Program Facilitator	4	Facilitator survey	Individual interview
24	Key Stakeholder	4	Snowballing	Individual interview
25	Program Facilitator	5	Direct invitation	Individual interview
26	Coordinator	5	n/a	Meeting 1
Policy makers				
27a	Senior Project Officer	n/a	Direct invitation	Individual interview
27b	Senior Project Officer	n/a	n/a	Meeting 1
27c	Senior Project Officer	n/a	n/a	Meeting 2
28	Senior Project Officer	n/a	Direct invitation	Individual interview

1–28: each number represent a different participant, a,b,c,d: Represents a different occasion of data collection from the same participant

### Data collection

Data were collected from January to June 2015 and included 18 individual semi-structured interviews, a focus group in one area and observation of two meetings involving researchers, policy makers and implementers (Table 1). In total 28 individuals contributed to the data collected consisting of research staff (*n* = 4), policy makers (*n* = 2) and implementers (*n* = 22) from across five prevention areas. Implementers included program coordinators (*n* = 5) responsible for overall implementation of the Program in each area, program facilitators (*n* = 8) who delivered the Program to parents, and local stakeholders (*n* = 9).

The interviews and focus group guides were informed by the consolidated framework for advancing implementation science [31] which integrates existing implementation theories into five key domains shown to be critical to implementation success. Key topics covered included:

- Role in implementing the InFANT Program
- Planning for program implementation - who, how and why the InFANT Program was selected?
- Models of implementation, including any adaptions to the Program and degree of program fit with existing services/programs and policies.
- Support for implementation, including on the ground logistical support, management support, researcher and policy support
- Implementation challenges
- Perceived outcomes including program strengths and weaknesses
- Perceived sustainability of program and factors influencing sustainability
- Key lessons for researchers, implementers and policy makers

Individual interviews were conducted over the phone by RL and lasted 40 min on average (range:17 to 65 min). The focus group was conducted face to face, facilitated by RL and lasted one hour and five minutes.

Opportunistic data collection also occurred at two meetings of local implementers, research staff and policy personnel. These meetings provided an opportunity to gather data on key issues relating to the implementation of the Program. The first meeting lasting 1 h and 5 min involved seven individuals including representatives (*n* = 5) from all the prevention areas as well as research staff (*n* = 1) and policy personnel (*n* = 1). This meeting focused on models for implementation where participants discussed implementation progress, challenges and various program adaptions that had been made. The second meeting, lasting 1 hour and 35 min included representatives (*n* = 3) from two prevention areas, research staff (*n* = 2) and policy personnel (*n* = 1). The focus of this meeting was to share the preliminary findings from the interviews and focus group to firstly verify key themes arising and secondly, to use this to generate further discussion and insights about key implementation issues.

### Data analysis

The interviews, meetings and the focus group were audio recorded with participants’ permission and transcribed verbatim. Transcripts were imported into Nvivo 10 which was used for coding, sorting and retrieval of data. The study used thematic analysis informed by the methods of Braun and Clarke [32] and involved the following steps undertaken by RL:

1. *familiarisation with the data* by checking the accuracy of transcripts against audio recording.
2. *line by line coding of the data* using an inductive approach guided by the research aims, resulting in the development of an initial coding framework. The coding framework was iteratively refined based on new concepts identified in the data.
3. *review of codes to identify broader conceptual themes*. At this stage the researchers’ knowledge of empirical literature and existing frameworks for implementation science helped in conceptualising codes into broader categories.
4. *Review of all data within a given theme* to identify common and divergent views using constant comparison technique [30].
5. *First draft of results section* which involved re-reading data coded at each theme, along with memo’s about sub-themes to succinctly summarise the theme using illustrative quotes.
6. *Member checking* which involved 1) presentation of key findings at a meeting of study participants (see above), which contributed to further data collection and elaboration and refinement of themes; 2) emailing coordinators (*n* = 3), research staff (*n* = 2) and policy personnel (*n* = 1) a copy of the initial draft results section of the manuscript. Participants were asked to comment on whether the themes sufficiently captured their views on the key factors influencing translation process and whether any key issues were omitted in the representation of the results. There was strong agreement with the themes presented and only minor modifications were made to the results based on feedback from participants.

### Reflexivity and the role of the researcher

As the study methods were informed by a constructivist approach, it is important to make explicit the role and background of the researcher which may influence how one understands and interprets the data [30]. RL undertook all data collection and analysis with member checking and input into final results. RL is a postdoctoral researcher with experience in research translation having worked on previous empirical studies exploring the process of research translation [33–37] and conceptual studies [5, 38]. Over the past 18 months, RL had been engaged with the researchers, implementers and policy makers involved in the translation of the Program. This has provided in-depth understanding of the translation issues where RL has essentially been a participant observer. We believe this adds strength, heightening RL’s sensitivity to translation and implementation issues in the data. RL was not involved in the original InFANT Program trial and had no input into the development of the Program.

## Results

### Description of study sites

All five of the eligible prevention areas took part in this study and had been implementing the Program between six and 15 months. All areas were in the lowest tertile for socio-economic disadvantage as defined by an area level indicator of disadvantage (Socioeconomic index for areas, [39]). Four of the areas were located in metropolitan Melbourne, with populations ranging from 25,000 to 150,000. One area was in a regional location in Victoria, approximately 550 km from Melbourne with a population of around 50,000. This is in contrast to the areas participating in the RCT of the Program where only three of 14 areas were in the the lowest tertile for socioeconomic disadvantaged (eight medium, three high) and all areas were located within metropolitan Melbourne [21].

### Role of researchers, policy makers and implementers

Researchers, policy makers and implementers all had specific roles to play in the scaling up and translation of the InFANT Program into routine practice (Table 2). For researchers, this centered on translation of intervention materials from the research trial into a ‘pre-packaged program’ ready for community wide delivery, developing and delivering a training program to implementers as well as being an expert point of contact for the Program. Policy makers provided support towards the translation effort by funding prevention areas as well as working closely with these areas to support multiple system wide prevention strategies including the implementation of specified programs. The Department also funded the printing of InFANT Program materials and funded researchers’ involvement in translation activities. Policy makers also acted as an important link between researchers and implementers on the ground. Local implementers were responsible for all aspects of program delivery at the community level including program planning and adaption, engaging delivery agents/services and program evaluation.

**Table 2:** The role of researchers, policy makers and local implementers in the translation process and key barriers and facilitators

Role/Translation tasks	Facilitators of translation	Barriers to translation
Researchers		
• Negotiate funding for translation activities • Translate research intervention into pre-packaged program materials • Develop and deliver training program for community facilitators • Develop and maintain program website and update program materials as required • Meetings with actual and potential program delivery agents • Point of contact for expertise on the Program for policy makers and practitioners • Custodian of program intellectual property • Advice on program sustainability and evaluation	• Well established relationships between researchers and policy makers • Funding of researcher involvement in translation activities • Researcher/institutional commitment to research translation • Coordinating role for research translation activities • Intervention program initially designed to be scalable and feasible to implement within existing service delivery structures.	• Staff turnover amongst policy makers • Researcher capacity – translation not core business • Translation activities not traditionally rewarded for researchers • Funding for researcher involvement in translation time limited
Policy makers		
• Funding prevention areas • Funding researchers involvement in translation activities • Selection of evidence based programs for implementation • Facilitate meetings and connections between implementers and researchers • Printing and distribution of program materials	• Prevention funding available for 12 prevention areas • Personnel available to support program implementation • Well established relationships with researchers and program implementers • Awareness and knowledge of the InFANT Program throughout the research phase • The InFANT Program addressed a gap and aligned with policy context of using programs within a complex systems approach	• Changes in government and funding arrangements • Resistance to programs as part of a systems based approach to prevention
Local implementers		
• Plan program delivery • Engage delivery partners, • Program administration, delivery and evaluation	• Prevention area funding • The InFANT Program addressed a gap in services • Access to a pre-packaged evidence based program • Program training and local mentoring • Access and support from researchers who designed the Program • Strong partnerships with delivery agents • Coordinators / champions/leadership • Congruence with existing programs, services and policy • Program adaption to suit local context	• Heavy administrative burden of the Program • Availability of workforce to deliver the Program • Competition with existing programs (one area) • Lack of centralised program evaluation

### Key factors affecting the translation process

A number of key barriers and facilitators to the translation process were identified (summarised in Table 2) and these are outlined in the following key themes.

#### Program specific factors

A number of program specific factors were considered important in influencing program uptake both at the policy and local community level. At the policy level, the feasibility of program delivery was considered to be critical in selection of the Program for dissemination. Researchers reported that the Program was designed with scalability and community implementation in mind. The process evaluation spoke to the feasibility of the Program in terms of delivery within the MCH setting as well as high rates of recruitment and retention. The positive outcomes of the research trial in terms of maternal beliefs, attitudes and behaviours were also considered important in policy makers endorsing the Program, despite the trial not demonstrating changes in the primary outcome of child weight:

*“the primary outcome was weight and we didn’t change weight. But…I’ve promoted fairly strongly to the Department of Health (‘the Department’) that we’ve seen changes in maternal attitudes and beliefs, …a number of the mediators, if you like, have improved…They were very impressed by the fact that 87 percent of people we approached wanted to participate, which I guess evokes some maternal interest in the space…That groups have worked quite well, so seven out of ten people attended four of six sessions. The mechanics appear to be a good model that they were interested in” (Research staff 1).*

At the local level, the InFANT Program was perceived to address an area of need and a gap in current service provision as there were very few (if any) existing healthy lifestyle programs targeting parents with young infants. Much of the current focus was on preschools and schools. The Program content including the clarity of the Program key messages, the discussion based nature of the groups as well as mode of delivery through engaging families, all appealed to local implementers. There was a general endorsement of the concept of starting healthy lifestyle messages early to prevent future problems and associated impact on service demands. The fact that the Program was ‘pre-packaged’ including program resources and training made it an attractive option as discussed by this local implementer:

*“because it was a pre-packaged program all the resources were there, the training was offered …so we identified a need, identified where we wanted to run it, found brilliant staff to run it, got the okay from sort of management level and then we were able to just kind of…take it up as a whole and roll it out (Coordinator 5d).*

Prevention areas had direct access to research staff involved in the design of the InFANT Program through face to face meetings, a dedicated training program and an online forum. This was considered to be an important facilitator for local implementation as described by this participant:

“*one of the main factors for encouraging uptake was the support that was delivered around it,…there were only two programs where the department engaged the researchers themselves to support the implementation. So that was in my view a big bonus for the HTCs (prevention areas) just because they got access to the researchers, they felt quite supported” (Policy maker 28).*

#### Partnerships

Strong partnerships were an important facilitator at every stage in the translation process. Researchers actively sought to engage policy makers early in the research phase of the InFANT Program. This well-established trusted relationship facilitated the early sharing of findings from the trial and helped to establish the researchers’ expert role and credibility. However, the high turnover of policy staff was a barrier to continuity of partnerships.

*“I think what the existing relationship did was gave us the opportunity to get our foot in the door, so we were a trusted source, we were considered to be experts in the field” (research staff 1).*

At the local level, the viability and sustainability of delivering the Program was dependent on engaging key delivery partners including MCH services, local council and community health services. The funded prevention workforce played a critical coordination role in engaging these partners during the initiation of the Program. A key engagement strategy included aligning the goals of the Program with the needs and priorities of key stakeholders. This involved using a combination of ‘bottom up approaches’ such as informal conversation with individual staff and ‘top down approaches’ such as negotiation between high level managers and formal agreements.

“*it's having a workforce that is able to do a lot of that legwork and, yeah, pitch it in such a way that a director or a manager can say, “Okay, I see our stake in this program” (Key stakeholder 13).*

It was acknowledged that building such partnerships takes time, requires a dedicated staff member(s) to drive partnership development as well as clear goals, regular communication and mutual benefits. A number of contextual factors were noted to influence partnership development across the areas involved, including the size of the area (with partnerships easier to forge in smaller areas with fewer stakeholders and existing relationships), individual personalities (degree of openness to innovation and willingness to champion the Program), whether prevention staff were co-located with key partners and the degree of program fit with the local policy context.

#### Coordinators, champions and leaders

Coordinators, champions and leaders were key drivers in the translation of the InFANT Program into routine practice and these individuals worked at the local level as well as in policy and research. As mentioned above, Prevention Coordinators were critical in bringing key partners around the table to agree on how the InFANT Program would be delivered at the local level. They did much of the ‘leg work’ of setting up the administration of the Program, reducing the risk for partners because Prevention Coordinators took initial responsibility for managing the Program.

*“Often you can have different people across different organisations, teams, departments, that are interested but you need someone to kind of bring it together, set it up” (Coordinator 5d).*

Prevention Coordinators who had already begun implementation of the Program often became champions and advisers to other areas considering taking the Program on. Individual MCH nurses and managers engaged in delivering the Program also became champions in promoting the Program within and outside their own service.

Further, individual policy personnel played an important coordinating role in bringing together researchers and local implementers to learn from each other as discussed above. Having a policy position responsible for the implementation of the Program was considered important in ensuring this level of coordination occurred between researchers and local implementers. At the researcher level, having university staff responsible for coordinating contracts with the Department and other administrative tasks was important in allowing the lead researcher to focus on key translation tasks such as engaging with local implementers.

#### Workforce capacity

Workforce capacity to support the implementation of the Program at the community level and to support the translation effort more broadly was a major theme arising. At the local level, program adaption and strong partnerships were considered essential in ensuring a sustainable workforce to deliver the Program. In contrast to the research trial, in which dietitians were used to deliver the Program all areas (except one) adapted the Program to use MCH nurses and parent support workers to deliver the Program. In these areas, mentoring support was provided by local dietitians following the formal training program. This program adaption was predominantly influenced by workforce capacity and cost, as well as fit with the existing health professional role.

“*You have to adapt it to what you’ve got capacity to run. We’ve run it with Maternal and Child Health nurses and parent support workers which makes it much more cost effective” (Coordinator14a)*.

MCH nurses and parent support workers were considered a natural fit for the role because of their well established and credible relationship with parents and because they already addressed issues related to infant feeding and active play in their routine consultations. The InFANT Program was considered an innovative way of more comprehensively addressing topics already covered in individual consultations, potentially saving consultation time and costs. As such, two areas reported embedding program delivery into existing MCH nurse and parent support worker roles, as a way of making program delivery more sustainable. Two areas had specifically funded the time of MCH nurses and parent support workers to deliver the Program as part of a pilot. The area that chose to use dietitians to deliver the Program perceived their expert skills and knowledge to be a selling point for getting parents to the Program. However the dietitians admitted themselves, that due to their limited capacity, additional support is likely to be needed in the future. More recently other allied health staff and health promotion officers in the area have been trained to provide ‘back up’ support to dietitians delivering the Program if required.

At the local level, the administration of the Program including organising groups, booking venues and facilitators, was a major burden largely taken on by Prevention Coordinators. To ensure sustainability of program administration in the absence of ongoing prevention funding, Coordinators discussed a number of options. These included making further adaptions to the Program to reduce the administrative burden, seeking funding for administrative support or trying to embed these tasks in an existing administrative role which would depend on strong partnerships with other services.

“*So it just seems like the weaknesses might be linked to the admin/workforce intensity of the Program.. if you don’t have other partners engaged, then that might be a little bit more difficult” (Policy maker 27a).*

Workforce capacity to support program translation was also an issue from the researcher and policy maker perspective. Research staff commitment to ‘make a difference’ was an important driver of their involvement despite having limited capacity to undertake research translation activities which are not considered core business or traditionally rewarded in academic roles.

*“It's so tough because it's so much an add-on to my core business, which is ironic isn’t it when you think that the academic role of developing knowledge and capacity, and then when it comes to actually doing what we said we wanted to do, which is implement it, we really had very little capacity to do that” (Research staff 1).*

Furthermore university systems may not be set up to easily support program delivery such as accepting payment for program training or the storage of program resources. Research staff suggested that alternative models for research translation are needed within the university sector such as dedicated funds and staff for research translation and researchers undertaking secondments to service delivery settings to support program roll out. At the policy level, staff capacity was also an issue with staff cut backs making it difficult to have a dedicated position to support program implementation.

#### Context – Fit with existing policy, program and services

The extent within which the InFANT Program fitted with the broader policy environment, as well as local programs and services was a central theme influencing translation opportunities. At the conceptual level, there was a strong tension between the Department’s focus on system level approaches to prevention with an emphasis on creating supportive policies and environments for behaviour change on the one hand, and programs, such as the InFANT Program, focusing on individuals/families on the other. This tension was resolved at the local level by demonstrating how individual programs, like the InFANT Program, fitted within and complemented a system based approach*:*

*“If you’re going to get the biggest bang for your buck I think you’d want to make sure that it’s [the Program] linking into things …we thought, well, if parents are getting this information early they’re going to be more receptive to that information when settings and services are taking that up in kinder and long day care…thinking about it broadly and where it fits into the broader system is important too” (Coordinator 22a).*

As discussed previously, the InFANT Program fitted well with the priorities and focus of local MCH services in most areas and this was essential for partnering with this service to deliver the Program. The Program was a ‘natural’ fit with first time parent groups running in all areas. These groups formed the basis of recruitment to the Program and provided a mechanism of linking parents to other services and programs locally, an important priority of both MCH services and local council. However, in one area, MCH services were running other concurrent parenting programs which were perceived to be competing with the InFANT Program for nurse and parent attention. The same area reported that initially it was difficult for MCH Service to be engaged as priorities and resources for the service had already been allocated on the basis of previous annual council business plans:

*“we’re operating in the local government context, it is a challenge to implement things in a timely manner when there are plans and strategies …they go over a four year period and there is already resources allocated and prioritisation that’s occurred…So for example, you know, an Infant type program wasn’t necessarily a priority with Maternal and Child Health.” (Coordinator 17a).*

#### Balancing adaption versus program fidelity

As expected, all areas had adapted or proposed to adapt the Program to some extent from the original trial (Table 3). Most common changes included choice of program facilitator, recruitment methods and use of local supplementary materials. Adaptions were largely driven by the need to make the Program viable to deliver in terms of staffing costs as well as to improve the fit with existing programs and services, to increase program reach and tailor to specific population needs. There was a clear tension between the need to adapt the Program to fit the local context and the need to maintain program fidelity and integrity as articulated by this participant:

**Table 3:** Summary of actual or proposed adaptions and rationale

Adaption	Rationale	Area
Program material supplemented with local information/resources	• Tailor to needs of local population (culturally and linguistically diverse groups) • Provide information on local services/facilities	All areas
Use Maternal and Child Health nurses or parenting workers to deliver the Program (instead of dietitians as per the trial)	• More cost effective • Limited capacity of dietitians • Good fit with existing role of MCH nurses and parenting workers	1,2,3,5
Recruit parents outside of first time parent groups	• To increase recruitment to the Program and reach	1,3,4,5
Reducing the number of sessions from 6 to 3 or 4.	• To reduce burden on facilitators and make the Program more viable to run • To fit with existing first time parent groups and individual Maternal and Child Health nurse consultations	2, 5
Plan to offer age specific group sessions open to anyone	• Reduce administrative burden of recruiting and following up multiple groups over time • Open up the Program to more parents potentially improving reach	1, 2
Amalgamate some groups for sessions beyond 12 months of age	• To address lower retention rates amongst parents with older babies • To make groups viable, limited capacity of facilitators	4
Key program messages were integrated into an existing program format	• Successful existing program already underway	5

Area 1,2,3,5: metropolitan Melbourne, Area 4 regional Victoria

*“…we’ve got a program that is being developed on good evidence, it’s been demonstrated to be effective in this way…And then there’s reality and our ability to kind of change and adapt the Program for what is realistic for us into the future, that will be challenging for us and getting support from [the university] around that” (Coordinator 17a).*

Implementers and policy makers called for clear guidelines from researchers about what were the core components of the Program required to maintain fidelity and effectiveness and what aspects could be adapted.

*“I guess the randomised control trials just cannot be repeated and used in a practical sense. So …making recommendations …what are the critical things that can't be changed, and what are some of the things that possibly could be changed or could be adapted to maintain integrity for those programs, and possibly testing them in that way as well.” (Policy makers 27a).*

While researchers were considered to have ownership over the Programs’ intellectual property, there was some discussion about the value of having a centralised evaluation framework to monitor both program fidelity and outcomes, recognising that programs do not operate in isolation but are part of the broader system wide prevention effort.

“*What would be really nice is to test some of this stuff and actually be able to …gather some information about what’s actually happening to the participants, and what benefits are the participants getting” (Research staff 2b).*

### Sustainability and scaling up

All the areas with the exception of area four (smaller rural area), chose to pilot the implementation of the Program in one or two neighbourhoods, or in one cohort of parents. The rationale for this was to test implementation processes and models and assess the feasibility and value of the Program before considering area wide dissemination. Three of the five areas largely embedded the delivery of the Program into existing roles within MCH or allied health services, with the view of setting up sustainability systems for program delivery beyond funding of prevention areas:

*“…we’re really trying to embed it in the work of Community Health. So if Healthy Together [prevention areas] is not refunded or it gets less funding that it continues, so it’s embedded within the Allied Health teams,…Child and Maternal Health structure as well, so if we are to pull away that it continues “ (Coordinator 22a).*

In contrast, two areas directly funded program delivery with the view of using the pilot phase to make a business case for continued funding through local council or other external sources.

“*So what we are hopeful [of] is that if we can have enough evidence and information that can create a very strong case for council and for budgets and for Maternal and Child Health nurses, the value of this. Then it may be sustained” (Coordinator 17a).*

Local implementers recognised the challenge of obtaining ongoing funding for the Program and hence were exploring options to further integrate the Program into existing service delivery structures, roles and existing programs.

“*I think as a program by itself, I don’t know how financially and time-wise it can expand without combining it with other programs that are already there*” (Program Facilitator 25).

There was general agreement that further program adaptions were likely to be required to better integrate the Program into what was feasible locally. Adaptions being considered included running age specific group sessions open to anyone (rather than existing parent groups), reducing the number of sessions or delivering the Program via existing groups such as playgroups.

From a policy perspective, there were varying views about program sustainability. One view was that state or federal funding of programs was unlikely, hence programs need to be embedded within a complex system based approach to prevention that provided an infrastructure to promote sustainability and scaling up.

“*I mean there's not going to be funding available to support and fund multiple programs, I doubt in the future to come…So it will just be about how that program can be integrated within the system” (Policy Maker 27a).*

Another view was that the InFANT Program should be a universal program with re-current government funding as part of an ongoing commitment to prevention in the early years:

*“… [the Program] needs to be funded by government and delivered by local council. The Government should make a commitment to early childhood and the InFANT program is one of those things…It’s not fair that some local councils implement and others don’t, …it should be universal” (Policy Maker 28).*

From the research staff perspective, maintaining and updating the Program website and materials and providing training to local implementers were ongoing activities that would require research staff time and hence funding. The short term nature of funding for researchers to be involved in these translation activities was a barrier to sustained program implementation.

## Discussion

This study provides new insights into the respective role that policy makers, researchers and implementers play in the translation of a health promotion program into practice and factors influencing this process. As discussed below, a number of key themes were identified by these stakeholders as being important in the translation of the InFANT Program from research to community level implementation.

In line with the findings from this study, the evidence of program efficacy or effectiveness has been found to be only one of many intervention characteristics influencing research uptake [6, 13, 31, 33, 40]. Other important factors identified in previous implementation research include, the credibility of the Program source, its feasibility, the quality of program materials, program adaptability, trialability, relative complexity and cost [31]. Our findings concur with recent research highlighting the importance of end users having information on program reach and costs, key service delivery issues such as acceptability and fit of the interventions with existing delivery models, to help inform decisions about the scaling up of public health interventions [40]. It also suggests the importance of researchers designing scalable and feasible interventions from the outset that align with the policy context. This is likely to require co-development of programs with practitioners on the ground or extensive formative work, as was the case with the InFANT Program [15, 23, 24]. This underscores the importance of researchers conducting rigorous process evaluations as part of efficacy /effectiveness trials to inform external validity of public health interventions, an area generally poorly reported by researchers [17, 19, 35].

The tension of balancing program adaption against maintaining program fidelity identified in this study, has been a commonly reported theme in the dissemination of research interventions into practice [13, 41–44]. While greater program fidelity has been shown to be associated with better outcomes [45], adapting intervention programs to better suit the needs and circumstances of local communities has also been shown to be essential for successful sustained implementation [8, 41, 42]. To help find this balance between fidelity and adaption, implementation researchers have proposed various guidelines [41, 46]. At the heart of these recommendations are the need for researchers to clearly articulate the Program’s core components based on a logic model of how the intervention is proposed to work, ideally supported with mediation analysis to identify the ‘active ingredients’ of the intervention. This is then married with consultation with community implementers to refine and modify non-core components to ensure program fit with local circumstances in an iterative fashion [46]. It is also recommended that adapted programs are evaluated to assess effectiveness and that these steps ideally involve consultation between program developers and implementers [46]. Systems such as licensing agreements or program guidelines may be helpful in monitoring program fidelity and adaptions and encouraging consultation between researchers and implementers. Service delivery organisations (not for profit or commercial) may have a role to play in ‘rolling out’ evidence based public health programs and monitoring program quality and fidelity. An example of this is the DECIPHer Impact, a not for profit organisation set up to license the 'ASSIST' peer-smoking intervention to schools to ensure fidelity [47].

For researchers, avenues need to be explored to fund research translation activities, including initial consultation with communities around program adaptions and ongoing program support, such as the provision training and updating of program materials. This remains a challenge, with translation activities not traditionally part of research grant proposals when the study outcomes are not yet known. More recently, specific research translation grants have become more common and this may provide an avenue for researchers to work with community partners to fund such activities. In the absence of external funding, researchers are likely to need to consider program licensing fees to cover costs associated with supporting program delivery, as has been done with other widely implemented programs such as the Standford Chronic Disease Self Management Programs [48]. At a system level, there is likely to be more incentive for researchers to focus on translation in the future with a growing focus on measuring research ‘impact’ as part of the assessment of universities research outputs. In some countries, research ‘impact’ is being link to university funding, for example, in the UK Research Excellence Framework.

Strong partnerships between researchers, policy makers and implementers, as well as local partnerships were identified as critical to the translation of the InFANT Program into practice. Partnerships between researchers and end users of research, such as policy makers and practitioners, has been consistently shown to be a facilitator of research use and scaling up in previous empirical studies [8, 11, 12, 33, 37]. As with this study, engaging end users from the inception of a project and forging ongoing relationships with policy makers and practitioners has been shown to be important in promoting the uptake of health promotion programs [33, 37, 49]. In this study, engaging practitioners in program modification ensured that the Program was designed to fit existing service delivery structures (MCH services), was relevant to the policy focus on obesity prevention in the early years, all of which were important facilitators of program uptake. The implementation of the Program was facilitated by local implementers having direct access to researcher staff who designed or delivered the Program to provide training and guidance for implementation. Mentoring programs where researchers and practitioners can learn from each other during the translation process have been shown to be useful for both parties [50]. However, challenges remain in funding researcher time, with research translation activities often considered outside of the traditional academic role. Dedicated funding for research translation and the establishment of mentoring or secondment opportunities for researchers, policy makers and practitioners to work together may be a useful step forward in supporting partnerships between researchers and end users of research.

The findings highlight the importance of having key individuals responsible for driving and coordinating research translation across the domains of research, policy, and practice. This case study is unique in that funding was provided to support the translation efforts. At the local level, the prevention workforce as part of the local prevention infrastructure was critical in engaging and working with key partners to deliver the Program. These coordinators undertook critical research ‘translation activities’ including exploring how the Program fitted with and enhanced existing services and how it could be best adapted to ensure sustained program delivery. In the scaling up of public health programs, consideration needs to be given to who will undertake these research translation activities at the local level. From a researcher perspective, research staff need funding to support the translation of research interventions into pre-packaged programs ready for community wide implementation. This study also demonstrates the important role that policy personnel can play in supporting research translation, highlighting the value of incorporating this component into existing policy positions. Previous case studies of research interventions with positive practice and policy impacts [33] as well as a recent literature review of facilitators of scaling up [8], have demonstrated the importance of leaders, champions and coordinators in advocating for and supporting adoption of public health interventions into practice.

The study findings point to important lessons regarding the scaling up and sustainability of program implementation. It is yet to be seen whether the InFANT Program can be scaled up and delivered on an ongoing basis in the absence of funding from the Department for the Program. Given that ongoing funding for any program at a state or local level was unlikely at the time of this study, it appears that embedding program delivery into existing service infrastructure will be critical for both scaling up and sustainability of the Program. With the strong competing demands on practitioner time and resources, it will be important that ongoing program evaluation be conducted to support a business case for continuation of the Program locally within existing services. This points to the importance of cost effectiveness analysis to be conducted as part of intervention trials to help make the case for investing in particular intervention programs. This is in line with a recent narrative review, which identified establishing monitoring and evaluation systems and costing and economic modeling of intervention approaches as important success factors for scaling up public health interventions [8]. Alignment of the Program to both state and local policy context will also be important in harnessing ongoing support for the Program.

This study has a number of strengths and limitations. The strengths include the use of multiple varied case studies and cross case comparisons to explore the important influence of local context on implementation. We did however, only have one rural site participate in the study and the inclusion of additional rural sites may have resulted in different implementation issues emerging. We also did not include sites that chose not to implement the Program, which may have provided interesting additional insights into factors influencing initial uptake. Within cases, we interviewed a range of stakeholders including coordinators, program facilitators and those involved in supporting program delivery. There was variation however in the number of people interviewed across sites ranging from nine participants (site one) to two participants (site five) and this may have limited insights gained from some sites. The inclusion of researchers and policy makers involved in supporting program delivery as participants in the study was also a strength as it enabled a comparison of views across roles, providing important new insights from these various perspectives. While the study used a range of data collection methods including individual interviews, focus groups and recording of meetings involving key players, additional methods such as observation of program sessions, analysis of key documents and interviews with parents may have yielded additional insights. While all data was collected and analysed by a single researcher (RL), the trustworthiness of the findings was verified by participants on two separate occasions and the role and background of the research was made explicit.

## Conclusion

This study highlights the important and complementary role that policy makers, researchers and practitioners can play in the translation of health promotion programs into routine practice. New avenues need to be explored to comprehensively bring together researchers and end users at all phases of the research to practice continuum. This is likely to lead to the development of more feasible and scalable programs, assist in adapting programs to fit local circumstances, while maximising program fidelity and supporting implementation at the local level. Key recommendations for researchers arising from this study include the need to develop feasible and scalable interventions from the outset; to incorporate comprehensive process evaluation measures, including cost effectiveness to inform future program roll out; and to identify core program components that are important for program fidelity. Consideration should also be given to additional mediation and dose response analysis to be conducted to inform program fidelity recommendations. Recommendations for practitioners include the importance of having a key individual responsible for coordinating translation activities in the initial startup phase, engaging key delivery partners to enable the Program to be adapted to fit and become part of existing local service delivery infrastructure. Consideration needs to be given to ongoing program evaluation at the local level to help create a business case for sustained program delivery. At the policy level, funding for research translation activities and partnerships between researchers and end users needs to be built into existing research funding schemes.

## Additional file

Additional file 1: Healthy Living Program and Strategy selection criteria. (DOCX 199 kb)
