# Supplementary material for: Translating an early childhood obesity prevention program for local community implementation: a case study of the Melbourne InFANT Program
Source: BMC Public Health. 2016 Aug 8;16:748. doi: 10.1186/s12889-016-3361-x (PMC4977772; doi:10.1186/s12889-016-3361-x)
Supplement: Additional file 1: — Healthy Living Program and Strategy selection criteria. (DOCX 199 kb) [file 12889_2016_3361_MOESM1_ESM.docx]

**Healthy Living Programs and Strategies Selection Criteria - Background**

As part of Victoria’s prevention effort, a healthy living program and strategy (HLPS) quality assessment process was developed to provide an opportunity for local prevention areas to implement quality health promotion programs and strategies. The selection criteria built upon existing tools such as the Gippsland Region’s evidence health promotion resource and the Commonwealth’s Healthy Living Network.

A centralised and systematic process was needed to avoid duplication of effort in assessing programs as too often local areas have been undertaking this process in isolation.

The selection criterion is outlined below:

**The Healthy Living Programs and Strategies Selection Criteria**

1. The program’s objectives are consistent with/contribute to one or more of the overarching HLPS aims to:

- increase healthy eating, physical activity and/or healthy weight; and
- decrease smoking and/or harmful/hazardous consumption of alcohol (for adults) by targeting groups or communities and providing:
- information or education increasing self-efficacy and health literacy
- practical skills development;
- participation opportunities, contributing to increased healthy eating, physical activity and/or healthy weight;
- programs to strengthen community action / create supportive environments

1. Programs and strategies are consistent with Healthy Food Charter, [National Nutrition](http://www.nhmrc.gov.au/guidelines/publications/n29-n30-n31-n32-n33-n34) and [Physical Activity](http://www.health.gov.au/internet/main/publishing.nsf/Content/health-pubhlth-strateg-phys-act-guidelines) guidelines
2. There is evidence of program effectiveness*
3. Cost effectiveness of the program has been considered (where available)*
4. There is evidence of adequate participation and sustained benefit
5. The program is inclusive of identified priority population groups
6. The program can be adapted to local circumstances and needs
7. There is no additional cost to participants for the purchase of specific foods or supplements
8. Participation costs or charges (if required) are reasonable for the service provided. Low income families / communities are not disadvantaged by cost.
9. Programs and strategies designed and/or promoted for weight loss meet the also meet the criteria in Box 2

* **Evidence of program effectiveness and cost effectiveness will take into account:**

1. Number of studies

2. Research design of studies (should be the highest level appropriate)

3. Quality of studies

4. Consistency of findings

It is acknowledged that programs may not fully meet all selection criteria. The aim is to select the best programs, based on available evidence, relevant to community need and priorities. Gaps in evidence (for selected programs) should be addressed with the inclusion of appropriate evaluation processes designed to fill gaps and inform future program development and delivery.

**Box 1. Additional criteria for programs and strategies designed and/or promoted for weight loss (NICE 2016)**

- help people assess their weight and decide on a realistic healthy target weight (people should usually aim to lose 5–10% of their original weight)
- aim for a maximum weekly weight loss of 0.5–1 kg
- focus on long-term lifestyle changes rather than a short-term, quick-fix approach
- are multi-component, addressing both diet and activity, and offering a variety of approaches
- use a balanced, healthy-eating approach
- recommend regular physical activity (particularly activities that can be part of daily life, such as brisk walking and gardening) and offering practical, safe advice about being more active
- include some behaviour change techniques, such as keeping a diary and advice on how to cope with ‘lapses’ and ‘high-risk’ situations recommend and/or provide ongoing support

NICE, 2016

*NICE 2016. Obesity: Guidance on the prevention, identification assessment and management of overweight and obesity in adults and children. London: National Institute of Health and Clinical Excellence. Downloaded from www.nice.org.uk/nicemedia/live/11000/30365/30365.pdf*

A list of recommended programs and strategies was created to fast track and support local prevention areas in their decision making about what to implement within their communities. Infant was one of these programs.

Over time, the selection criteria was refined to allow for innovation and experimentation. This included a process for developing ‘safe to fail’ programs and strategies. This process is demonstrated in Figure 1.


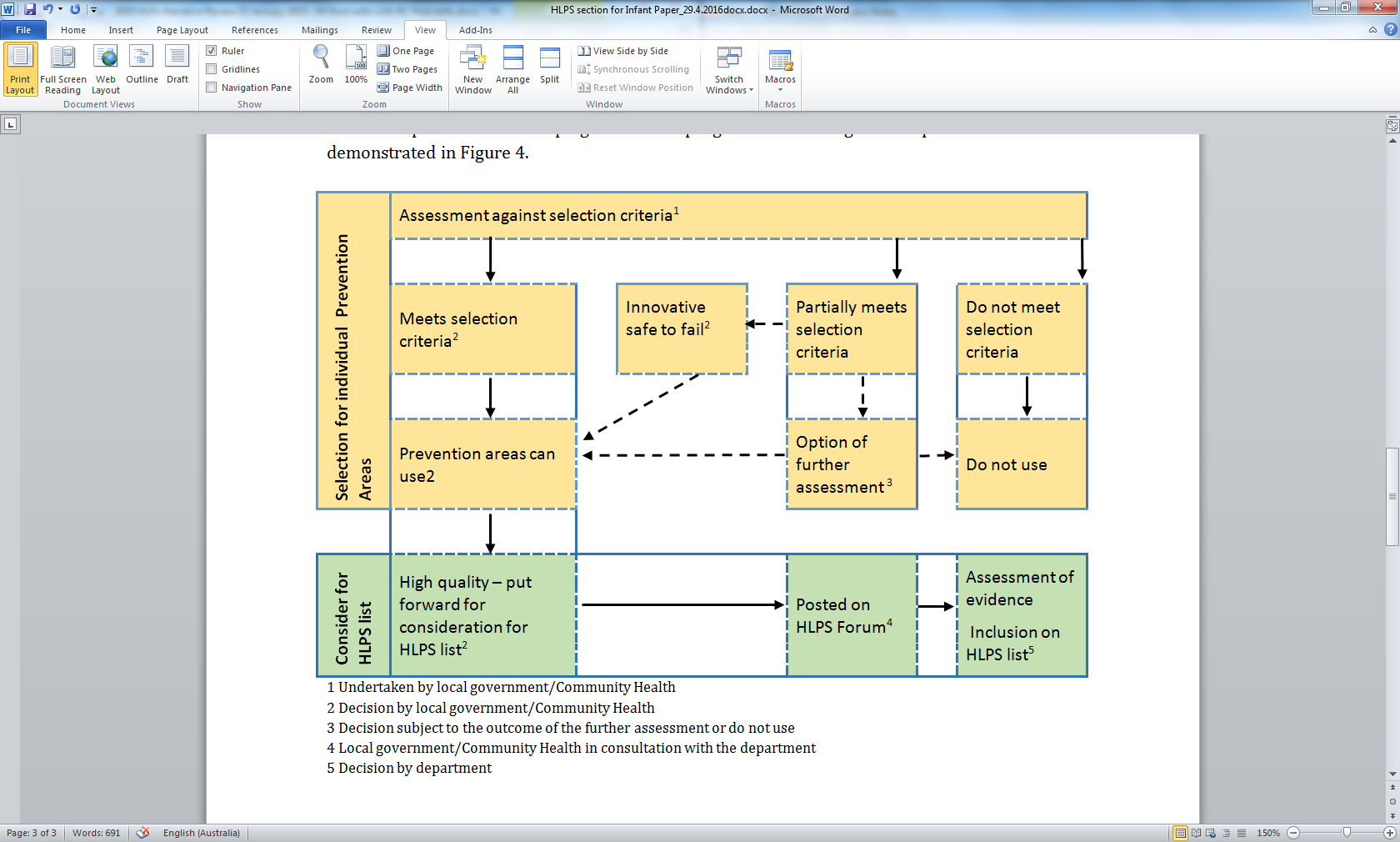


**Figure 1. Process for developing ‘’Safe to Fail’’ programs**
